# Tonically Active α2 Subunit-Containing Glycine Receptors Regulate the Excitability of Striatal Medium Spiny Neurons

**DOI:** 10.3389/fnmol.2017.00442

**Published:** 2018-01-09

**Authors:** Svetlana M. Molchanova, Joris Comhair, Deniz Karadurmus, Elisabeth Piccart, Robert J. Harvey, Jean-Michel Rigo, Serge N. Schiffmann, Bert Brône, David Gall

**Affiliations:** ^1^Laboratory of Neurophysiology, ULB Neuroscience Institute, Université Libre de Bruxelles (ULB), Brussels, Belgium; ^2^Biomedical Research Institute, University of Hasselt (UHasselt), Hasselt, Belgium; ^3^School of Health and Sport Sciences, University of the Sunshine Coast, Sippy Downs, QLD, Australia; ^4^Sunshine Coast Health Institute, Birtinya, QLD, Australia

**Keywords:** dorsal striatum, medium spiny neurons, glycine receptors, tonic current, excitability

## Abstract

Medium spiny neurons (MSNs) of the dorsal striatum represent the first relay of cortico–striato–thalamic loop, responsible for the initiation of voluntary movements and motor learning. GABAergic transmission exerts the main inhibitory control of MSNs. However, MSNs also express chloride-permeable glycine receptors (GlyRs) although their subunit composition and functional significance in the striatum is unknown. Here, we studied the function of GlyRs in MSNs of young adult mice. We show that MSNs express functional GlyRs, with α2 being the main agonist binding subunit. These receptors are extrasynaptic and depolarizing at resting state. The pharmacological inhibition of GlyRs, as well as inactivation of the GlyR α2 subunit gene hyperpolarize the membrane potential of MSNs and increase their action potential firing offset. Mice lacking GlyR α2 showed impaired motor memory consolidation without any changes in the initial motor performance. Taken together, these results demonstrate that tonically active GlyRs regulate the firing properties of MSNs and may thus affect the function of basal ganglia.

## Introduction

The striatum is the primary input site of the basal ganglia, a set of brain nuclei, important for voluntary movements and motor learning (Graybiel, 2008; Doyon et al., 2009). It collects the incoming information from cortical areas in order to build motor patterns based on the current environmental situation and past experience. This information is transferred along the thalamo-cortical loops as a direct output to different brainstem motor centers. As a result of information processing along this neuronal network, basal ganglia direct the initiation of the desired movement and inhibit competing movement patterns (Cui et al., 2013).

Medium spiny projection neurons (MSNs) represent 95% of all neurons in the striatum (Kreitzer, 2009). They form two distinct types, defined by their innervation targets. Striatonigral (STN) MSNs innervate the substantia nigra pars reticulata and the internal segment of the globus pallidus (or entopeduncular nucleus in rodents). Activation of this pathway leads to disinhibition of the basal ganglia output structures and participates in initiation of voluntary movement. Striatopallidal (STP) MSNs innervate the external globus pallidus, which inversely controls the same thalamo-cortical motor circuits and inhibits competing movements during the realization of the motor task.

Despite of different innervation targets and distinct protein expression profiles, MSNs of both types have similar morphological and electrophysiological properties (Kreitzer, 2009). They are medium-sized cells with extensive dendritic trees, covered with spines. MSNs are hyperpolarized at rest, with a resting membrane potential (RMP) of around -80 mV and a high intrinsic membrane conductance due to voltage-gated and inwardly rectifying potassium channels. STP MSNs are more excitable and receive slightly denser glutamatergic innervation than STN MSNs, but their RMP and characteristic firing pattern are similar (Kreitzer and Malenka, 2007).

Medium spiny neurons fire action potentials (APs) upon excitatory synaptic drive by corticostriatal afferents, and their activity is modulated by GABAergic and cholinergic inputs from local interneurons and dopaminergic projections from the substantia nigra pars compacta (Wickens and Wilson, 1998; Kreitzer, 2009). Although GABAergic transmission exerts the main inhibitory control of MSNs, they also express glycine receptors (GlyRs) – another type of ligand-gated chloride channels (Sergeeva, 1998). However, the subunit composition and functional significance of GlyRs in MSNs have not been described to date.

Glycine receptors are expressed throughout CNS of mammals, with varying expression density and cellular localization (Legendre, 2001). Activation of GlyRs leads to the opening of an anion-permeable channel and to a fast increase in chloride conductance. The role of these receptors is well characterized in the spinal cord and brainstem, where glycinergic interneurons regulate the generation of APs by motor neurons through glycinergic synapses, participating in regulation of breathing and pain sensitivity (Harvey et al., 2004; Manzke et al., 2010). GlyRs are also expressed in other brain structures, including the cerebral cortex, the hippocampus, and the ventral tegmental area; and may be located postsynaptically (Dumoulin et al., 2001; Husson et al., 2014), presynaptically (Kubota et al., 2010; Xiong et al., 2014), or extrasynaptically (Mori et al., 2002; Wang et al., 2005; Salling and Harrison, 2014; McCracken et al., 2017), depending on the cell type. There are four agonist-binding subunits of GlyRs (α1–4, encoded in mice by *Glra1–4*), and one structural subunit (β, encoded by *Glrb*). The main agonist-binding subunits in the adult brain are GlyR α1 and α3, whereas α2 modulates brain development and gradually disappears after birth (Legendre, 2001; Avila et al., 2013a; Morelli et al., 2017). Mutations in *GLRA1* underlie development of hyperekplexia – a rare congenital human motor disorder (Shiang et al., 1993); several mutations in *GLRA2* have been linked to autism spectrum disorder (Piton et al., 2011; Pilorge et al., 2016; Zhang et al., 2017), and *GLRA3* has been implicated in the pathogenesis of temporal lobe epilepsy (Eichler et al., 2008).

In the striatum of adult rodents, there is expression of α2 and β subunits of GlyRs (Jonsson et al., 2012). Glycinergic currents were demonstrated in MSNs and cholinergic interneurons (Sergeeva, 1998; Darstein et al., 2000). Parvalbumin- and calretinin-positive interneurons in the human striatum also express GlyRs (Waldvogel et al., 2007). Functional studies have shown that GlyRs inhibit glutamatergic (Adermark et al., 2011) and activate dopaminergic (Yadid et al., 1993; Darstein et al., 1997) synaptic transmission. A recent study found tonically active GlyRs in MSNs from dorsal striatum and nucleus accumbens (McCracken et al., 2017). Despite this, there is no data on the expression, functional properties, and biological roles of GlyRs in the dorsal striatum. Here, we confirm the presence of functionally active GlyRs in MSNs of the dorsal striatum and determine the identity of the α-subunits of these receptors. We show that GlyRs are tonically active and depolarizing, regulating the active membrane properties of MSNs. In addition, we demonstrate impaired motor learning in mice lacking the GlyR α2 subunit. These data suggest that GlyRs act as a balancing force, and, together with potassium currents, determine the RMP and responsiveness to excitation of MSNs, which may be important for the proper motor learning and memory consolidation in basal ganglia circuitry.

## Materials and Methods

### Animals

C57/Bl6, Drd1-EGFP, Drd2-EGFP, and α2-knockout mice (*Glra2*-KOs) of the age of 8–16 weeks were used in the study. *Glra2*-KOs were generated by deletion of the exon 7 of *Glra2* (Avila et al., 2013b). Drd1-EGFP and Drd2-EGFP (Gong et al., 2003) mouse lines were obtained from Jackson Laboratory. *Glra2*-KOs were fully back-crossed to C57/Bl6, and Drd1a-EGFP and Drd2-EGFP to CD1 background and genotyped as described previously (Gong et al., 2003; Avila et al., 2013b). C57/Bl6, Drd1-EGFP, and Drd2-EGFP mice of both sexes and *Glra2*-KO and WT males were used in the study.

All experimental procedures were performed according to the Institutional Animal Care Committee guidelines and were approved by local ethics committees. All efforts were made to minimize the suffering and number of animals used.

### Immunostaining and Microscopy

For GlyR and CTIP2 immunostaining, C57/Bl6 mice were deeply anesthetized with avertin (2,2,2-tribromoethanol 1.25%; 2-methyl-2-butanol 0.78%, 20 μl/g, ip, Sigma–Aldrich) and transcardially perfused with 0.1 M phosphate-buffered saline (PBS), followed by paraformaldehyde (PFA, 4% in PBS). Brains were post-fixed in PFA overnight, and 50 μm coronal slices were done on a vibratome. Immunostaining using pan-GlyR antibodies (mAb4) and rat anti-CTIP2 was performed according to the following protocol (Rousseau et al., 2012). After wash in Tris-buffered saline (TBS; 0.05 M Tris and 0.9% NaCl, pH 8.4) with 0.05% Tween, slices were processed in tri-sodium citrate (10 mM) for 20 min at 95°C. Aldehyde groups were removed by incubation in sodium borohydride (0.2% in TBS) and slices were post-fixed in 100% methanol (-20°C, 20 min) to inhibit endogenous peroxidase activity. After that, slices were incubated for 1 h in blocking solution (10% normal donkey serum, 0.2% Triton X-100 in TBS) and then overnight with the following primary antibodies: (mouse, Synaptic Systems, 1:100) and anti-CTIP2 (rat, 1:200, Abcam, United Kingdom). Primary antibodies were revealed by incubation with secondary antibodies coupled to either Alexa Fluor 488 (donkey anti-mouse, 1:500, Life Technologies) or Alexa Fluor 555 (donkey anti-rat, 1:500, Life Technologies). Control immunostainings were performed without primary antibodies; no non-specific signal was observed. Slices were mounted using hardset DAPI (Vectashield) for counterstaining.

In some *in vitro* electrophysiology experiments, for verification of the cell type, biocytin (0.5%) was included in the filling solution. After recordings, electrodes were gently removed from the cell and slices were transferred into 4% PFA for overnight fixation. To reveal the biocytin staining, slices were permeabilized in 0.1% Triton X-100 in PBS for 2 h and then incubated for another 2 h with streptavidin-NL557 (1:2000; NL999, R&D Systems). To stain nuclei, slices were then incubated with Hoechst 33342 (1:5000, Invitrogen) for 10 min and mounted using Fluorsave mounting medium (Calbiochem).

Images were acquired on a Laser-Scanning Confocal System 228 (LSM 780; Zeiss, Oberkochen, Germany), mounted on an Axio observer Z1 inverted microscope (Zeiss, Oberkochen, Germany). Argon laser (488 nm), He–Ne laser (543 nm), and a 405 nm blue laser diode were used for excitation of Alexa Fluor 488, Alexa Fluor 555, and Hoechst, correspondingly, and emission signals were filtered. C-Apochromat 40×/1.1 NA water-immersion objective was used for acquisition. Images were acquired using Zen 2010 Software (Zeiss, Oberkochen, Germany) with resolution 14.5 pixels/μm and processed in Fiji software (Schindelin et al., 2012).

### Quantitative Real-Time PCR of GlyR Subunit mRNAs

Relative expression of GlyR subunit mRNAs in dorsal striatum was evaluated using C57Bl/6 mice. One sample contained tissue material from one animal. Dorsal striatum was dissected from whole brain and was mechanically homogenized in Qiazol Lysis Reagent (Qiagen). Total RNA was extracted using RNeasy Minikit (Qiagen). One microgram of purified total RNA was used to synthesize a first strand cDNA, using MMLV-RT Kit (Invitrogen) and random hexamer (Roche). QPCR analysis was performed on 10 ng of cDNA with Power SYBR green (Applied Biosystems) on ABI 7500 Fast Real-Time PCR System (Applied Biosystems). Primers for *Glra1*, *Glra2*, *Glra3*, *Glra4*, and *Glrb* were from Qiagen (QT00172221, QT00132020, QT02325316, QT00146972, QT00162911). Primer efficiency was tested using a serial dilution protocol, and reached values between 94% and 103%. Ct (threshold cycle) values for each gene were normalized to Ct values of RPL13 (Forward: 5′-CCCGTGGCGATTGTGAA-3′, Reverse: 5′-TCATTGTCCTTCTGTGCAGGTT-3′) and RER-1 (Forward: 5′-CCACCTAAACCTTTTCATTGCG-3′, Reverse: 5′-TTTGTAGCTGCGTGCCAAAAT-3′). The *Glra2* values were used as a reference and set at an arbitrary value of 100, and all other values were calculated as percentage of *Glra2* expression.

### Preparation of Acute Slices

Animals were anesthetized with halothane and killed by decapitation. Brains were removed and 220 μm coronal slices, containing cortex and striatum, were made using a vibratome (Leica VT1000S) in ice-cold dissection solution (mM: 140 choline Cl, 2.5 KCl, 1.25 NaH_2_PO_4_, 7 MgCl_2_, 0.5 CaCl_2_, 26 NaHCO_3_, 10 glucose; equilibrated with a 95% O_2_ and 5% CO_2_). Slices were transferred to the recovery chamber with recording artificial cerebrospinal fluid (ACSF, see the composition below) with elevated concentration of MgCl_2_ (to 3 mM). After the initial recovery period of 30 min at 32°C, slices were stored at room temperature and used 1–4 h after cutting.

### *In Vitro* Electrophysiology

For electrophysiological recordings, slices or isolated neurons were placed in a submerged chamber with a volume of about 1 ml. Slices were superfused with ACSF containing (in millimolar): 127 NaCl, 2.5 KCl, 1.25 NaH_2_PO_4_, 1 MgCl, 26 NaHCO_3_, 15 D-glucose, and 2 CaCl_2_; 5% CO_2_–95% O_2_, at a rate of 1–2 ml/min. All recordings were done at 32°C. MSNs within the dorsal striatum were identified with a 63× water immersion objective of a Zeiss Upright Microscope (Axioskop 2FS Plus; 140 Zeiss, Oberkochen, Germany). The cell type was confirmed by presence of EGFP fluorescence, the pattern of the evoked APs, or by imaging of biocytin-filled cells.

Cells were recorded in whole-cell configuration, using borosilicate-glass patch electrodes (Hilgenberg GmbH, Malsfeld, Germany) obtained with a vertical two-stage puller (PIP 5, HEKA Elektronik). Pipette resistance was typically 4–7 MΩ. All recordings were obtained by EPC-10 amplifier (HEKA Elektronik). For whole-cell recordings, data were sampled at 10 or 20 kHz with gain 1 mV/pA and filtered with in-build low-pass filter at 2.9 kHz. Cells were first recorded in voltage-clamp mode at holding potential of -80 mV, as previously described (Gall et al., 2005). Series resistance was monitored by injection of 10 mV voltage steps, and if the change in series resistance during the experiment exceeded 30%, the recording was discarded. PatchMaster Software (HEKA Elektronik) was used for data acquisition. Recordings were not corrected for liquid junction potential, and series resistance was not compensated for. Offline analysis was done using IgorPro Software (WaveMetrics) with Patcher’s Power Tools and NeuroMatic plugins and Microsoft Excel software.

*Glycine evoked currents and dose–response curve.* Cells were recorded in voltage-clamp mode, using the filling solution of the following composition (high-chloride filling solution, in millimolar): 120 CsCl, 0.022 CaCl_2_, 4 MgCl_2_, 10 HEPES, 0.1 EGTA, 5 lidocaine *N*-ethyl chloride, 5 Na_2_-phosphocreatine, 4 MgATP, and 0.5 Na_2_GTP. Increasing concentrations of glycine were applied with Perfusion Fast-Step System (Warner Instruments, Hamden, CT, United States). Positioning of the tubes for fast application was verified by application of 30 mM KCl. Evoked glycinergic currents were recorded in presence of 10 μM gabazine, 10 μM CNQX, 0.1 μM DHβE, 5 μM L-689,560, and 1 μM tetrodotoxin (TTX). Amplitude of the currents was analyzed in IgorPro, and dose-dependent response curve was fitted with Hill equation, using the build-in fitting function in IgorPro software, which utilizes minimal Chi-square algorithm.

*Endogenous glycinergic currents* were assessed by patch-clamp recordings in whole-cell configuration, using high-chloride filling solution. mIPSCs were recorded in voltage-clamp mode at -80 mV in presence of 10 μM AMPA receptor blocker CNQX, 0.1 μM nicotinic receptor blocker DHβE, NMDA receptor blocker 5 μM L-689,560, and voltage-gated sodium channel blocker 1 μM TTX with or without 10 μM of GABA_A_ receptor blocker gabazine. The duration of the recordings was 5 min for each condition. Tonic glycinergic current was estimated as a change in holding current of the cell, patched with high-chloride filling solution and kept at -80 mV, upon application of 1 μM strychnine. After the baseline period of 1 min, strychnine was fast-applied and the holding current was recorded for another 5 min. Mean values of holding current during the first and sixth minutes of recording were measured. As a control, we recorded the baseline current in the same experimental setup, applying ACSF without any inhibitors through fast-perfusion system. In another set of experiments, effect of strychnine was evaluated in the presence of 1 μM TTX. In these experiments, TTX was applied through fast-perfusion system during 1-min baseline and 5-min strychnine application.

*Reversal of glycinergic currents* was evaluated by direct application of 3 mM glycine to the cell, recorded in perforated patch configuration. The composition of the filling solution was 125 mM KMeSO_3_, 13 mM KCl, and 10 mM HEPES, pH 7.2. Gramicidin was added from DMSO stock to the filling solution, resulting in a final concentration of 0.1–0.2 mg/ml. Access resistance was monitored till it reaches at least 70 MΩ (conductance around 14 pS). RMP was recorded in current clamp mode. Then, recordings were performed in voltage clamp, with changing holding voltage from -90 to -30 mV by 10 mV steps to assess the reversal potential of glycinergic currents. Glycine was applied by Perfusion Fast-Step system. Obtained values were not corrected for liquid junction potential, which was 13.4 mV.

*RMP and intrinsic excitability* of MSNs were recorded in whole-cell configuration and current clamp mode, using filling solution, where [Cl^-^] was set to give *E*_Cl_ = -60 mV. The composition of filling solution was as follows (in millimolar): 115 KMeSO_3_, 7 KCl, 0.022 CaCl_2_, 4 MgCl_2_, 10 HEPES, 0.1 EGTA, 5 Na_2_-phosphocreatine, 4 MgATP, and 0.5 Na_2_GTP, pH 7.2. RMP was recorded without injecting any background current. AP firing was evoked by current injections, increased in steps of 10 pA, starting from the holding current, needed to keep the cell at -80 mV. Voltage values were not corrected for liquid junction potential, which was 13.3 mV. In experiments with acute strychnine application, due to the possible rundown of the excitability related to the perfusion of the cytosol with filling solution, RMP and AP recordings were performed on separate control cells and cells pre-incubated with 1 μM strychnine for 15 min. In the latter case strychnine was also present in the perfusion medium.

### Behavior

Behavioral experiments were conducted during the light phase of the light–dark cycle with 4-month-old WT and *Glra2*-KO males. Experimental protocols were the same, as described previously (Chen et al., 2014; Lambot et al., 2016). The illumination in the room was set at 20–30 lux. For open-field test, animals were placed in a square open field (40 × 40 cm) for 1 h during three consecutive days. The motion of the animal was tracked with overhead video camera (Ethovision XT; Noldus Information Technology), and the distance, traveled during the session, was reported. The rotarod apparatus (accelerating model, Ugo Basile) was used in accelerating rotarod test. Animals were placed on a rod, rotating with the constant speed (4 rpm), and then, the rod was accelerated to 40 rpm over 5 min. Latency to fall was measured. Animals performed four trials per day during five consecutive days. For every day, the values from four trials were averaged for every animal. During single-pellet reaching test, animals were trained to grab the food pellets with one forepaw, and the accuracy of movement was estimated. Vanilla pellets (Dustless Precision Pellets^®^ Rodent, Purified, Bioserv) were used as a food source during experimental trials. Animals were food-restricted, starting 2 days before the experiment, to reduce the weight till 85–90% of the initial body mass by the date of training. After 2 days of habituation in the experimental cage for 20 min per day, for the next 2 days animals were trained to reach for pellets, located outside of the cage in a tray, by extending one forelimb through the narrow slit. During this shaping period, the animal was taught the location of the pellets, and dominant limb was determined for every animal. After that, during the experimental period, mice were trained to reach single pellet, located outside the cage on a fixed point, using the dominant limb, and the accuracy of the movement was evaluated. The length of the day session was 20 min or 30 attempts to reach the pellet. Results are presented as a percent of precise reaches out of all reaching movements during one daily session, for eight consecutive days.

### Statistics

Data are presented either as mean ± SEM, or as median, maximum, and minimum of the sample distribution, for a number of variables indicated for each experiment. Statistical analysis was done using Prism Software (GraphPad). Two independent groups of variables were compared using Mann–Whitney *U*-test and dependent variables were compared by Wilcoxon matched-pairs signed-rank test. In case of repeated measurements, data were analyzed by two-way ANOVA.

## Results

### Functionally Active Glycine Receptors Are Present in MSNs of the Dorsal Striatum

**FIGURE 1:**
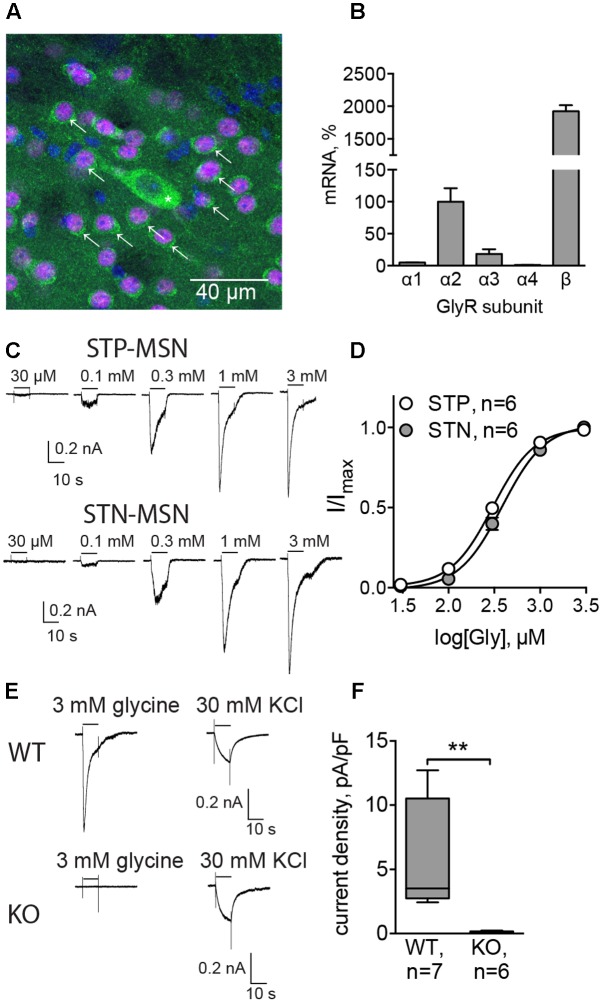
Functionally active glycine receptors (GlyRs) are present on MSNs. **(A)** Co-staining for GlyRs (mAb4, green) and CTIP2 (specific marker of MSNs, magenta). Cell nuclei are stained with Hoechst (blue). Some MSNs are indicated with arrows, and a cholinergic interneuron, identified by size, is indicated by an asterisk. **(B)** Quantitative PCR for α1, α2, α3, α4, and β subunits of GlyRs in dorsal striatum (*n* = 3 samples/3 animals). **(C)** Example traces of glycinergic currents in STP and STN MSNs, recorded with high-chloride filling solution in the presence of gabazine, CNQX, DHβE, L-689,560, and TTX. These recordings were made in Drd1-GFP and Drd2-GFP mice. **(D)** Concentration–response curve of the evoked glycinergic currents in STP and STN MSNs (STP MSNs: 6 cells/5 animals; STN MSNs: 6 cells/5 animals). No significant difference was found between currents, recorded from STP and STN MSNs. **(E)** Example traces of glycinergic currents (3 mM glycine) and KCl-evoked currents in MSNs from WT and *Glra2*-KO (KO) mice. **(F)** Quantification of current density, evoked by 3 mM glycine, in WT and *Glra2*-KO MSNs (WT: 7 cells/4 animals, KO: 6 cells/4 animals, ^∗∗^*p* < 0.01).

The presence of GlyRs in MSNs was evaluated by immunolabeling of dorsal striatum of adult C57Bl/6 mice, using pan-glycinergic antibodies and CTIP2 as a specific marker of MSNs (Arlotta et al., 2008). We found that 91 ± 2.5% of CTIP2-positive cells co-localized with GlyR signal (eight sections from two animals, **Figure 1A**), which confirmed the expression of GlyRs by MSNs. Quantitative PCR on mRNA extracted from dorsal striatum of adult C57Bl/6 mice demonstrated the expression of GlyR α2, α3, and β subunit transcripts. When the expression of GlyR α2 was taken as 100%, the expression of GlyR α3 was 18.4 ± 7.3%, and the expression levels of GlyR α1 and α4 were less than 5% (GlyR α1: 4.9 ± 0.3; GlyR α4: 1.0 ± 0.4; *n* = 3 samples/3 animals, **Figure 1B**). The expression of the structural subunit β was 20 times higher than the levels of α2 (1924.0 ± 93.1%).

To verify whether GlyRs expressed by MSNs are functional, we recorded the currents evoked by local application of increasing concentrations of glycine. STN and STP MSNs were identified by EGFP expression in Drd1-EGFP and Drd2-EGFP mice. In the presence of inhibitors of glutamatergic, cholinergic, and GABAergic transmission, glycine-evoked inward currents, which increased in a concentration-dependent way (*n* = 6 cells/5 animals in each group, **Table 1** and **Figures 1C,D**). These results are comparable with values in striatal slices (McCracken et al., 2017) and give us a higher boundary for the EC_50_ values, since the actual concentration of agonist reaching the cells in the slice is lower than the one in the perfusion pipette, due to diffusion. However, these data allow us to compare glycinergic currents in the two populations of MSNs. Although current density was higher in STN MSNs, this difference was not statistically significant, and other properties of the evoked glycinergic current were similar for STN and STP MSNs (**Table 1**).

**Table 1:** Characteristics of evoked glycinergic currents in STP and STN MSNs.

	STP MSNs (*n* = 6)	STN MSNs (*n* = 6)
EC50 (μM)	317.18 ± 25.40	411.11 ± 34.38
Hill coefficient	1.90 ± 0.11	1.91 ± 0.06
Maximal current density (pA/pF)	4.18 ± 1.44	6.45 ± 1.27

To evaluate the subunit composition of functionally active GlyRs, we applied 3 mM glycine onto MSNs in brain slices, obtained from a mouse line in which GlyR α2 subunit expression is ablated (*Glra2* knockout animals, *Glra2*-KOs) and used wild-type (WT) littermates as controls. *Glra2*-KO MSNs did not show any response to application of 3 mM glycine (*n* = 6 cells/4 animals), whereas MSNs from WT littermates responded to glycine application with prominent inward currents (*n* = 7 cells/4 animals, current density 5.54 ± 1.60 pA/pF, *n* = 7; **Figures 1E,F**).

Together, these data confirm that MSNs in the adult murine striatum express functional GlyRs, with α2 being the main agonist-binding subunit. Both populations of MSNs have similar expression levels of GlyRs, and for the following experiments, we did not distinguish between them.

### Glycine Receptors in MSNs Produce a Tonic Current, Dependent on the Presence of Electrical Activity in the Slice

**FIGURE 2:**
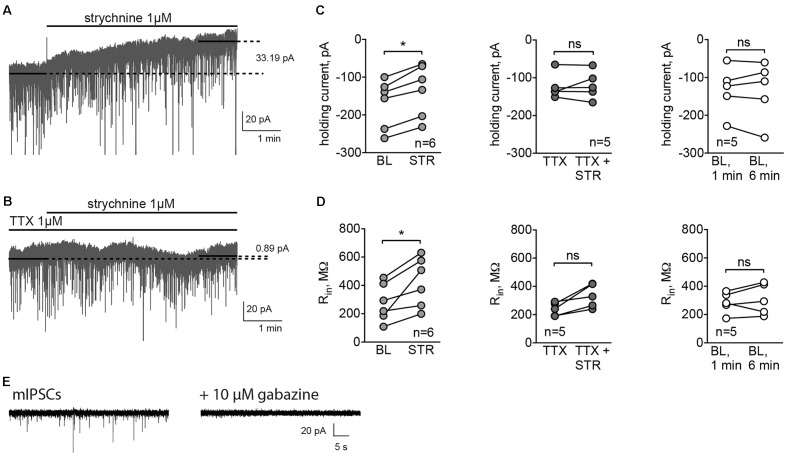
GlyRs in MSNs are tonically active. **(A,B)** Example traces of holding currents, recorded in whole-cell mode using high-chloride filling solution. After recording baseline current for 1-min without **(A)** or with **(B)** TTX (1 μM), we applied strychnine (1 μM) through fast perfusion system and recorded holding current for another 5 min. Mean holding current during baseline period was compared to the mean holding current during the last minute of the recording. **(C,D)** Values of holding current **(C)** and *R*_in_ **(D)** in experiments with application of strychnine with or without TTX, and control recordings, performed during application of ACSF without drugs through fast-perfusion system [BL (baseline): 5 cells/3 animals, STR (strychnine): 6 cells/5 animals, TTX: 5 cells/3 animals; ^∗^*p* < 0.05]. **(E)** Example traces of mIPSCs, blocked by application of 10 μM gabazine (3 cells from 3 animals recorded).

To check whether GlyRs are responsible for phasic synaptic currents, we recorded mIPSCs using high-Cl filling solution, and then inhibited GABA_A_ receptors to see whether gabazine-insensitive synaptic currents could be detected. In these experiments, C57Bl/6 mice were used, and the MSN cell type was verified subsequently by the characteristic morphology revealed by the biocytin labeling of the patched cell (data not shown). The amplitude of mIPSCs was 25.69 ± 3.31 pA, and the frequency 1.23 ± 0.11 Hz (*n* = 3 cells/3 animals). In the three experiments conducted, 10 μM gabazine blocked all synaptic currents (example traces are shown in **Figure 2E**), suggesting that GlyRs are not located in synapses. Therefore, to test the presence of extrasynaptic tonically active GlyRs, we recorded the holding current without any inhibitors, and then applied the GlyR antagonist strychnine. Strychnine (1 μM) reduced the holding current of MSNs (**Figures 2A,C**) by 30.95 ± 5.35 pA (*n* = 6 cells/5 animals, *p* < 0.05). Control recordings performed in the same conditions, but without strychnine application, display a stable holding current (change in the holding current 1.96 ± 9.13 pA, *n* = 5 cells/3 animals; **Figure 2C**). Interestingly, in the presence of the fast sodium-channel blocker TTX, strychnine did not change the holding current of MSNs (**Figures 2B,C**, change in the holding current 3.73 ± 8.27 pA, *n* = 5 cells/3 animals), which demonstrates that the release of the endogenous GlyR agonist is activity dependent. Strychnine also increased the input resistance (*R*_in_) of the MSNs. *R*_in_ was measured by the application of voltage steps before and after the recording of the holding current. Application of strychnine for 5 min increased *R*_in_ by 145.18 ± 37.78 MΩ (**Figure 2D**, *p* < 0.05). When the holding current was recorded in control conditions – without drug application, *R*_in_ did not change (**Figure 2D**). In the presence of TTX, a slight increase in *R*_in_ after strychnine application was observed, but the effect was not statistically significant (**Figure 2D**).

### Tonically Active Glycine Receptors Are Depolarizing

**FIGURE 3:**
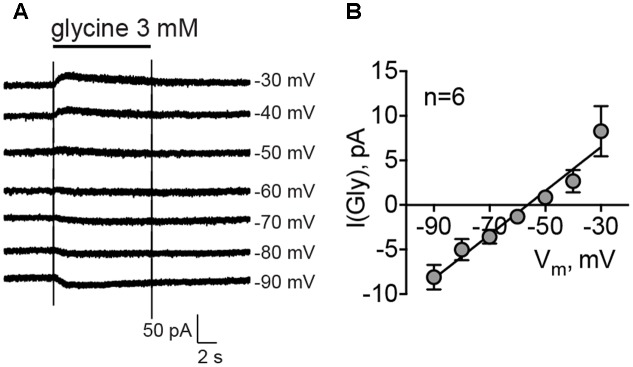
Reversal potential of glycinergic currents, recorded by gramicidin-perforated patch clamp. **(A)** Example traces of the glycinergic current, evoked by direct application of 3 mM glycine at different membrane potentials. **(B)** Mean values of glycinergic currents at each membrane potential (*n* = 6 cells/4 animals).

It has been shown previously that the reversal potential of Cl^-^ currents in MSNs is around -60 mV, which is more positive than the RMP for this cell type, -80 mV (Wilson, 2007). To evaluate the reversal potential of glycinergic currents in MSNs, cells were recorded using a perforated-patch approach with gramicidin, and 3 mM glycine was applied at different membrane potentials. The mean reversal potential of glycinergic currents, estimated by the linear fitting of the current amplitudes at different membrane potentials, was -54.42 ± 2.94 mV (**Figures 3A,B**, *n* = 6 cells/4 animals). The mean RMP in the same condition was -77.67 ± 0.44 mV.

**FIGURE 4:**
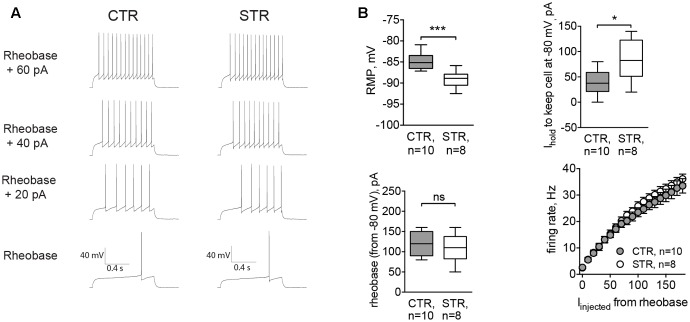
Acute inhibition of GlyRs affects RMP and the current needed to hold the cell at –80 mV (*I*_*h*old_). Recordings were done in whole-cell mode with [Cl^–^]*_i_* adjusted to set the reversal potential of Cl^–^ currents at -60 mV. APs were evoked by incremental current injections, when RMP of the cell was kept at –80 mV by constantly applying *I*_*h*old_. **(A)** Example traces of evoked APs in control conditions (CTR) and in the presence of strychnine (STR, 1 μM). **(B)** RMP, holding current, rheobase, and firing rate of MSNs with and without strychnine (CTR: 10 cells/5 animals, STR: 8 cells/4 animals; ^∗∗∗^*p* < 0.001, ^∗^*p* < 0.05). Current values for firing rates are plotted starting from the rheobase.

To assess the functional effect of this tonic current, we performed experiments where RMP and the intrinsic excitability of MSNs were recorded in control conditions and in the presence of strychnine. Here, cells were recorded in whole-cell configuration with the filling solution adjusted to give a reversal potential for Cl^-^ equal to -60 mV. The cell type was confirmed by the characteristic MSN firing pattern. In strychnine conditions, the cells were more hyperpolarized (-89.10 ± 6.70 mV, *n* = 8 cells/4 animals) compared to controls (-84.76 ± 6.59 mV, *n* = 10 cells/5 animals, *p* < 0.001; **Figure 4B**, RMP). Control values of RMP were more negative than those obtained by perforated patch recordings. The difference may be attributed to the effect of a cytosolic dilution by the filling solution. To record evoked APs, cells were brought to -80 mV, and the corresponding depolarizing holding current was significantly higher in strychnine-treated MSNs (39.38 ± 7.88 pA in control conditions and 82.50 ± 13.80 pA in the presence of strychnine; *p* < 0.05, **Figure 4B**, *I*_hold_), consistent with the observed hyperpolarizing effect of strychnine on RMP. Strychnine did not affect either the rheobase, measured from the membrane potential of -80 mV, or the firing rate of somatic APs evoked by current injection (**Figures 4A,B**, rheobase and firing rate). Voltage threshold of the AP firing was not changed in the presence of strychnine (-48.80 ± 1.44 mV in control conditions and -48.90 ± 1.10 mV in the presence of strychnine). In other words, after pharmacological inhibition of GlyRs APs were induced at the same membrane potential, but more current had to be applied to reach this potential, starting from RMP. Taken together, these data show that glycinergic currents in MSNs depolarize the cellular membrane and affect the offset of the evoked AP firing (the current intensity needed to shift the membrane voltage from baseline values to the AP threshold levels (Mitchell and Silver, 2003).

### MSNs of the Glra2-KO Mice Are More Hyperpolarized and Less Excitable

**FIGURE 5:**
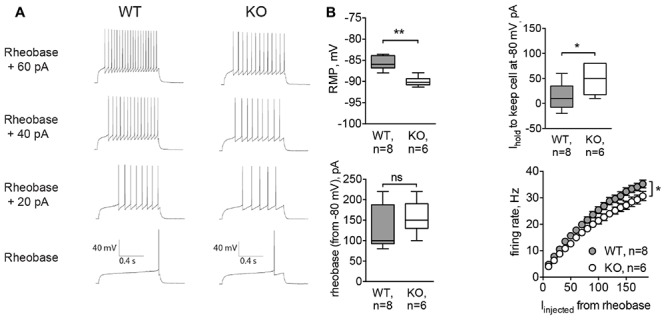
MSNs of *Glra2*-KO mice are more hyperpolarized and less excitable than WT cells. Recordings were done in the same configuration as in **Figure 4**. **(A)** Example traces of evoked APs in WT and *Glra2*-KO MSNs. **(B)** RMP, holding current, rheobase, and firing rate of WT and *Glra2*-KO MSNs (WT: 8 cells/3 animals; KO: 6 cells/3 animals; ^∗∗^*p* < 0.01, ^∗^*p* < 0.05).

To confirm results obtained by acute inhibition of GlyRs, we recorded MSNs from *Glra2*-KO mice. Similar to strychnine-treated cells, MSNs of *Glra2*-KO mice also had a hyperpolarized RMP (-89.99 ± 0.46 mV, *n* = 6 cells/3 animals, versus -85.62 ± 0.56 mV for wild-type littermates, *n* = 8 cells/3 animals, *p* < 0.01; **Figure 5B**, RMP) and needed higher values of depolarizing holding current to maintain a membrane potential at -80 mV (13.75 ± 9.34 pA in WT MSNs and 48.33 ± 12.22 pA in *Glra2*-KO MSNs, *p* < 0.05, **Figure 5B**, *I*_hold_). We also measured the intrinsic firing properties of MSNs from *Glra2*-KO and WT mice, and found that the rheobase was not affected, but the cells fired less APs in response to somatic current injection (*p* < 0.05, **Figures 5A,B**, rheobase and firing rate).

### Glra2-KO Mice Have Deficient Consolidation of Motor Memory without Apparent Changes in Locomotion

**FIGURE 6:**
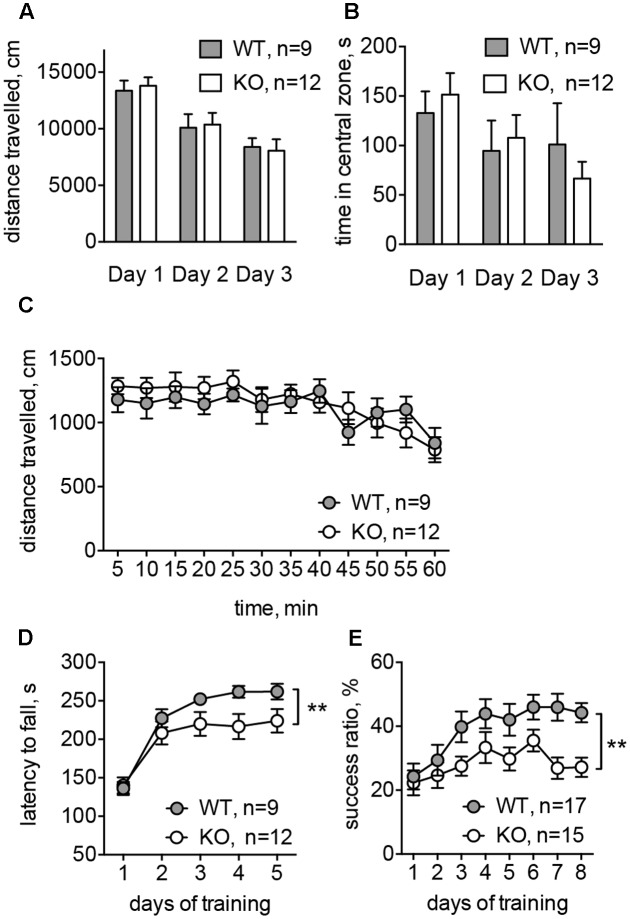
*Glra2*-KO animals displayed motor learning deficit without any changes in the basal motor performance. **(A)** Distance traveled during open-field test (1 h in three consecutive days). **(B)** Time spent in the central zone during the open-field test (per 1 h in three consecutive days). **(C)** Distance traveled during the first experimental day; data are presented per 5 min-bins. **(D)** Accelerated rotarod test. Data are from five consecutive days, with four trials during each day. **(E)** Single-pellet reaching test. After initial habituation and shaping phase (4 days) the success ratio of reaching a pellet was evaluated during 8 days (one session per day). *N* is the number of animals tested; ^∗∗^*p* < 0.01, two-way ANOVA.

To evaluate the basal ganglia-related behavioral performance in *Glra2*-KOs, we compared them to WT animals in a battery of behavioral experiments. In the open-field test, *Glra2*-KO animals behaved similarly to WT littermates. The distance traveled during 1-h sessions and the time spent in the central zone did not differ between the two experimental groups (**Figures 6A–C**). This indicates that basic locomotion, habituation, and level of anxiety are not affected in *Glra2*-KO animals. However, performance during motor learning tests was lower in *Glra2*-KOs, compared to WT controls. In the rotarod test (**Figure 6D**) and single-pellet reaching test (**Figure 6E**), the animals performed equally during the first day of motor learning. However, during subsequent days, *Glra2*-KOs demonstrated a shorter latency to fall in the rotarod test and a smaller number of successful reaches to the food pellet in the single-pellet reaching test (*p* < 0.01, **Figures 6D,E**).

## Discussion

The function of GlyRs in the brainstem and spinal cord has been studied in detail, while the presence and role(s) of GlyRs in the brain are only starting to be revealed. Previous data on the expression of GlyRs in the dorsal striatum are limited and inconsistent. While early *in situ* hybridization studies suggested that GlyRs were not present in the dorsal striatum (Malosio et al., 1991), subsequent immunostaining with pan-GlyR antibodies demonstrated that the expression of GlyRs is limited to cholinergic interneurons of the rat caudoputamen (Darstein et al., 2000). A later study in the human striatum added parvalbumin and calretinin-positive interneurons as cells expressing GlyRs (Waldvogel et al., 2007), but GlyR immunoreactivity in MSNs has never been detected. However, the presence of functionally active GlyRs has been suggested in MSNs of the rat striatum by recording agonist-evoked chloride currents (Sergeeva, 1998). These findings were confirmed recently both for dorsal striatum and nucleus accumbens (McCracken et al., 2017). Agonist-evoked glycinergic currents disappear in *Glra2*-KO mice, and tonic currents seem to be mediated by α3 subunit. However, as no glycine evoked currents were recorded in MSNs of *Glra2*-KO mice, the contribution of the α3 subunit to the tonic current in MSNs might be related to α3 expression in striatal interneurons or projecting terminals. Here, using immunostaining, qPCR, and electrophysiological recordings, we proved the presence of functional GlyRs in MSNs of adult mice. Immunostaining with pan-GlyR antibodies showed the presence of GlyRs in MSNs. This was confirmed by recordings of glycine-evoked chloride currents from both STN and STP MSNs. We also analyzed the expression of individual subunits of GlyRs in cDNA extracted from tissue preparation of dorsal striatum, and found that α2 and β subunits are predominantly expressed. By contrast, expression of the GlyR α3 subunit was much lower than that of α2, and probably corresponds to expression by interneurons (Sergeeva and Haas, 2001). Based on the expression pattern and the absence of any evoked glycinergic currents in MSNs of *Glra2*-KO mice, we conclude that GlyRs in MSNs are likely to be α2 homomers or α2β heteromers. These results add important information to our understanding of the GlyR subunit composition in the adult brain, since α2 was previously thought to be preferentially expressed during embryonic and early postnatal brain development (Avila et al., 2013a; Morelli et al., 2017).

Depending on the subunit composition, GlyRs may be located either extrasynaptically or synaptically, since the β subunit binds to gephyrin, which clusters GlyRs at postsynaptic sites (Legendre, 2001). We did not detect any phasic glycinergic synaptic currents, but strychnine induced a robust change in the tonic current. The onset of the change of this tonic current was slow, possibly due to the fact that strychnine is a competitive agonist and that it takes time to substitute the glycine molecules from the active site of the receptor (Legendre, 2001). The shape and temporal pattern of the change in holding current after application of strychnine was similar to that previously shown in cortical and hippocampal pyramidal cells and MSNs from dorsal striatum and nucleus accumbens (Zhang et al., 2008; Salling and Harrison, 2014; McCracken et al., 2017). These results support an extrasynaptic localization of these GlyRs, which are tonically active in the cortico–striatal brain slice, in accordance with earlier studies (Adermark et al., 2011; McCracken et al., 2017). A similar distribution of GlyRs has been shown in the hippocampus, where GlyRs do not contribute to synaptic transmission, but regulate the excitatory transmission and synaptic plasticity via tonic currents (Mori et al., 2002; Zhang et al., 2008; McCracken et al., 2017). Additionally, tonically active GlyRs have been found in the adult prefrontal cortex (Salling and Harrison, 2014; McCracken et al., 2017) and the ventral tegmental area of juvenile and adult mice (Wang et al., 2005).

Glycine receptors may be activated either by glycine or by taurine (Legendre, 2001). Taurine, but not glycine, is the endogenous ligand of extrasynaptic GlyRs in the hippocampus (Mori et al., 2002) and the ventral tegmental area (Wang et al., 2005) of adult animals, and, most likely, activates GlyRs during cortical development (Yoshida et al., 2004; Qian et al., 2014). In the rat striatum, immunostaining for glycine did not reveal any glycine-positive cell bodies and only sparse glycine-positive fibers (Rampon et al., 1996). The interstitial concentration of glycine, measured by *in vivo* microdialysis, is twofold lower than that of taurine in the rat striatum (Molchanova et al., 2004). Pharmacological modulation of neuronal activity affects the striatal release of taurine, but not glycine (Molchanova et al., 2004). Based on these findings, taurine, and not glycine, is the most likely endogenous ligand for GlyRs in striatum. However, there are no direct data on possible cellular sources of extracellular glycine or taurine in striatum, and more studies are needed to confirm the identity of the endogenous agonist of striatal GlyRs.

Tonic release of endogenous agonist(s) of GlyRs may be supported by several mechanisms, including spill-over from the synaptic cleft, leaking through membrane channels (volume-activated chloride channels and hemichannels) and reversal of carrier-mediated membrane transport (Molchanova et al., 2006; Harsing and Matyus, 2013). Although tonic release of either glycine or taurine occurs outside synapses, it may also be regulated by AP firing (Molchanova et al., 2006; Harsing and Matyus, 2013). We have shown that the release of endogenous agonist(s), activating GlyRs on MSNs, is TTX-sensitive. Although the exact release mechanism is not yet clear, these data propose that endogenous ligand of GlyRs is released from some neurons, which spontaneously fire APs in the corticostriatal slice, for example from the tonically active striatal interneurons.

MSNs of the dorsal striatum act as a high-pass filter for the incoming cortical signal. They are hyperpolarized at rest, and get activated only by a strong excitatory input (Wickens and Wilson, 1998; Kreitzer, 2009). Chloride channels play an important role in modulating the activity of MSNs, both via synaptic connections from interneurons and tonic GABAergic currents (Luo et al., 2013; Paille et al., 2013). The reversal potential of chloride currents mediated by GABA_A_ receptors in MSNs (shown to be -76 to -60 mV, Koos et al., 2004; Bracci and Panzeri, 2006) is more positive than the resting potential (-90 to -80 mV, Bracci and Panzeri, 2006; Gittis et al., 2010). Our data confirm that chloride currents through GlyRs have the same properties. In this case, chloride currents will depolarize MSNs when cells are most deeply hyperpolarized, and may, therefore, promote the escape of the membrane potential from hyperpolarization-activated potassium current (Wilson, 2007). Depolarizing phasic GABA_A_ transmission in MSNs converts subthreshold excitatory currents to suprathreshold ones, promoting AP firing and spike-timing-dependent plasticity at glutamatergic synapses (Bracci and Panzeri, 2006; Paille et al., 2013). Tonic GABA_A_ conductance, which is present preferentially in indirect pathway MSNs, lowers the rheobase of AP firing (Janssen et al., 2011). Our data strongly suggest that tonically active GlyRs may exert the same type of regulation of MSN firing and excitability.

Indeed, tonic GlyRs-mediated chloride conductance regulates RMP and the current needed for inducing AP firing in MSNs, thus influencing the offset of incoming excitatory signal and postsynaptic computation at the single-cell level. Strychnine did not change the firing frequency, but constitutive inactivation of the GlyR α2 subunit lowered the excitability of MSNs, thus affecting the gain of the transmitted signal. Modulation of neuronal offset and gain is important for maintaining the firing rate within the operational range (Mitchell and Silver, 2003; Semyanov et al., 2004). In the case of MSNs, this is important for overcoming tonic hyperpolarizing potassium currents and for being able to respond to the incoming excitatory stimulus.

A similar effect of depolarizing tonic chloride conductance has been observed in hippocampal interneurons, where tonic depolarizing GABA_A_ currents regulate the AP threshold and firing pattern (Pavlov et al., 2014). Keeping in mind the possible interneuronal origin of the endogenous agonist, we propose that tonically active GlyRs regulate the excitability of MSNs, providing positive feed-forward regulation of the cortico–striatal synaptic pathway.

Basal ganglia play an important role in motor learning and consolidation of the procedural memory. Striatal MSNs are the first relay of basal ganglia for the incoming cortical signal, and their activity is crucial for the realization of movement sequences and habit formation (Graybiel, 2008; Doyon et al., 2009). Our data obtained at the single-cell level strongly suggest that the regulation of the excitability of MSNs by GlyRs may be needed for the proper function of basal ganglia. Mutations in *GLRA2* are involved in the pathogenesis of autism spectrum disorder – a pathological condition characterized by deficits in social interaction and repetitive patterns of behavioral output (Piton et al., 2011; Fuccillo, 2016; Pilorge et al., 2016; Zhang et al., 2017). Impaired social behavior is traditionally linked to a deficient function of the cerebral cortex, whereas impaired motor performance, stereotyped routines, and repetitive movements may be the result of an abnormal function of the basal ganglia (Fuccillo, 2016). In this respect, the *Glra2*-KO mouse line might be utilized as an animal disease model to better understand the pathophysiology of autism at the cellular and network level.

*Glra2*-KO adult mice do not show any morphological changes in the brain, and the basal motor performance is not altered (Young-Pearse et al., 2006; Pilorge et al., 2016). However, deletion of *Glra2* causes a moderate microcephaly in newborn mice and disrupts the functional organization of the cerebral cortex (Avila et al., 2013b; Morelli et al., 2017), which corresponds to an impaired long-term potentiation in the prefrontal cortex and deficits in object recognition memory (Pilorge et al., 2016). We have shown that *Glra2*-KOs performed worse than WT littermates in motor learning tasks such as the rotarod test and the single-pellet reaching test. Performance in both motor learning tests used (especially in the single-pellet reaching test) is dependent on the function of the striatum (Hunter et al., 2000; MacLellan et al., 2006). Interestingly, performance of the animals during the first training session was not affected, which is in accord with previously published results (Pilorge et al., 2016). Novelty-induced locomotor behavior during first minutes of the trial, basic locomotion, habituation, and level of anxiety, evaluated in the open field test, also did not differ. However, motor skill development during subsequent experimental days of the motor learning tests was impaired. The observed changes may be related either to deficiencies in motor learning in *Glra2*-KOs, or to lower motor performance under pressure. The latter seems to be less likely, since we observed similar effects in two very different motor learning tests, involving different muscle performance and attention levels. These data underlie the importance of the GlyR α2 subunit for motor learning, and reveals further behavioral phenotypes in the mouse *Glra2*-KO. Based on the selection of the behavioral tasks and the low expression of the GlyR α2 subunit in other brain areas and cell types, we suggest the involvement of altered MSN function in the impaired performance of *Glra2*-KOs in the rotarod and single-pellet reaching tests. However, we cannot exclude the contribution of other brain areas, especially the motor cortex. We also cannot confirm that the changed RMP and firing offset of MSNs in *Glra2*-KOs is solely responsible for the altered function of these cells during behavioral performance. The absence of GlyR α2 subunit could affect the maturation of the synaptic circuitry leading to an impaired motor learning seen at the behavioral level.

Taken together, our results show that GlyRs in MSNs of the dorsal striatum are extrasynaptic and tonically active. Their activity is regulated by spontaneously firing neurons, possibly local striatal interneurons. When active, GlyRs stabilize the RMP and regulate the offset and, probably, gain of signal transfer along the basal ganglia network. A loss of the α2 subunit, and, consequently, of functional GlyRs in MSNs, leads to impaired memory consolidation during striatum-specific motor learning tasks. The study brings the understanding of the function of GlyRs in adult murine dorsal striatum, and this knowledge is important for future studies of disorders associated with these receptors.

## Author Contributions

SS, J-MR, BB, DG, and SM designed the experiments. JC, DK, EP, and SM performed the experiments. RH generated the *Glra2*-KO. JC, DK, and SM analyzed the data. SM wrote the article. All authors revised the article and agreed to be accountable for all aspects of the work.

## Conflict of Interest Statement

The authors declare that the research was conducted in the absence of any commercial or financial relationships that could be construed as a potential conflict of interest.
